# Effects of Smart City Policies on Green Total Factor Productivity: Evidence from a Quasi-Natural Experiment in China

**DOI:** 10.3390/ijerph16132396

**Published:** 2019-07-05

**Authors:** Baogui Xin, Yongmei Qu

**Affiliations:** College of Economics and Management, Shandong University of Science and Technology, Qingdao 266590, China

**Keywords:** smart city, green total factor productivity, difference-in-differences propensity score matching, quasi-natural experiment

## Abstract

When cities develop rapidly, there are negative effects such as population expansion, traffic congestion, resource shortages, and pollution. It has become essential to explore new types of urban development patterns, and thus, the concept of the “smart city” has emerged. The purpose of this paper is to investigate the links between smart city policies and urban green total factor productivity (GTFP) in the context of China. Based on panel data of 200 cities in China from 2007–2016 and treating smart city policy as a quasi-natural experiment, the paper uses a difference-in-differences propensity score matching (PSM-DID) approach to prevent selection bias. The results show: (a) Smart city policies can significantly increase urban GTFP by 16% to 18%; (b) the larger the city, the stronger and more significant this promotion.

## 1. Introduction

Urban growth is a global trend, the world’s urban population will be close to 70% by 2050 [1]. Following this world trend, urbanization is also developing rapidly in China. The urbanization level rose from 17.92% in 1978 to 54.7% in 2016, and will rise by 70% in 2030 [2]. However, China’s urbanization is mainly enacted through area expansion and population spatial agglomeration. This model will lead to population expansion, traffic congestion, resource shortages, and environmental degradation [3]. The 18th National Congress of the Communist Party of China clearly put forward the concept of “new urbanization”. Cities are implementing programs aiming to increase their sustainable development. These address several aspects, such as smart energy management, the increased use of renewables, and improvements in resource efficiency [4]. Smart city policy is an effective way to solve persistent sustainability issues in society [5]. The development and wide application of modern information communication technology (ICT) and internet technology provide the opportunity to base the smart city on these technologies, creating a model that meets development needs [6].

Many countries around the world have launched policy projects about the concept of Smart City. In November 2008, IBM first proposed the idea of “Smart Planet” and officially introduced the concept to the US federal government in January 2009, suggesting investment in building a new generation of intelligent information infrastructure. Singapore is one of the first countries in the world to establish a “smart country” construction strategy. Moreover, in 2006 and 2016, the “Smart Country 2015” plan and the “Smart State 2025” plan were launched in Singapore, respectively. In 2016, the Japanese cabinet first proposed the concept of “social 5.0” which is the ultra-smart society and the six necessary measures to achieve a super-intelligent society in the “Science and Technology Basic Plan”. These countries have made fruitful achievements in the construction of smart cities. China has begun to implement smart city policy in 2012.

Since the reform and opening up policy was implemented, China’s economy has maintained moderate-to-rapid growth. However, this growth has led to high resource consumption and severe pollution. China is an important case where increasing human activities are leading to environmental degradation [7]. To change this situation, the state proposed the concept of green development in the 13th Five-Year Plan. The plan also noted that the process and results of economic activities should be more “green” and “ecological” to achieve sustainable development in economic, social, and environmental terms. Total factor productivity (TFP) is an important economic concept for measuring the quality of economic development. With increasing environmental problems, the demand for energy savings and green adjustment is growing [8]. Green total factor productivity (GTFP) was proposed, which is to add environmental indicators to the calculation of TFP. It takes the constraints of energy input and pollution emissions into account [9]. Transforming the economic development mode and improving GTFP is an important way to realize sustainable development [10].

With the intensification of environmental protection and resource conservation, people are becoming more aware of GTFP. The public advocate the environmentally friendly economic growth mode and the concept of sustainable development. In-depth analysis of GTFP under environmental constraints is of considerable significance to find solutions to alleviate the contradiction between economic growth and environmental pollution [11].

Smart cities have become a popular concept because they have the potential to create a sustainable and livable urban future [12]. Given the accelerated growth of cities and the increasing demand for solutions to the negative impacts of urban development, researchers have become more interested in issues related to smart cities [13]. Recently, the theoretical research dynamics [14,15,16] and practical development status [17,18] of smart cities have attracted extensive attention from academic circles and industry internationally. There are also many documents examining the smart city policy from the perspective of planning and policy evaluation. Caragliu et al. found that smart city policy can promote urban innovation process through a general improvement of local knowledge production functions [19]. Yu et al. concluded that smart cities realize the interconnection and coordination of various subsystems through the collection, statistics, and analysis of information [20]. It helps the city system coordination and operation efficiently, reduce management costs, and improve resource allocation efficiency [20]. Through empirical researches, Shi et al. proved that the emission of pollutants in the region where smart city policy is implemented in China had decreased significantly [21]. GTFP is just related to these aspects, so we try to test whether or not a smart city policy can improve GTFP.

This paper aims to study the impact of smart city policies on GTFP from the perspective of an urban development pattern. Treating smart city policy as a quasi-natural experiment, the paper uses a difference-in-differences propensity score matching (PSM-DID) approach. The propensity score matching (PSM) method was first proposed by Heckman [22] to eliminate sample selection bias. The difference-in-differences method (DID) can solve the endogeneity problem and obtain the policy treatment effect. Subsequently, researchers continued to develop and combine the two to empirically study the policy effects [23,24,25]. They selected the control group through PSM and evaluated the impact of policies by using the DID analytic approach.

The paper is structured as follows. In Section 2, we give an overview of the theoretical background, including the smart city policy and research status, GTFP, and its calculations. In Section 3, we introduce research materials and methodology. In Section 4, we report experimental results and implement the robustness test. In Section 5, we analyze the experimental results and close the paper.

## 2. Theoretical Foundation

### 2.1. Smart City Policy

The concept of a smart city stems from the idea of the smart earth proposed by IBM in 2008. The smart earth is the product of digital cities and the Internet of Things. The construction of smart cities uses modern information technology to promote the interconnection, efficiency, and intelligence of urban operating systems, thereby creating a better life for citizens [26,27]. In short, the definition of “Smart City” is the use of information technology to attack urban problems.

In 2012, China officially issued the “Notice on the Pilot Work of National Smart Cities.” The first batch of smart city pilots involved 90 cities and county-level cities. In 2013, the Ministry of Science and Technology and the National Standardization Management Committee identified 20 cities including Qingdao and Jinan as “smart cities” technology and standard pilot cities (“smart cities” dual pilots). In 2014, with the approval of the State Council, the eight ministries and commissions including the National Development and Reform Commission, the Ministry of Public Security and the Ministry of Finance issued the “Guiding Opinions on Promoting the Healthy Development of Smart Cities”, requiring all regions and relevant departments to implement the tasks proposed in this guidance and ensure smart cities healthy and orderly advancement. The opinion also suggested that by 2020, several smart cities with distinctive characteristics will be built, and the gathering and radiation belts will be significantly enhanced.

Since the implementation of the smart city policy, it has penetrated all aspects of human production and life, such as smart medical care [28] and intelligent transportation [29]. In the process of building a smart city, various types of smart sensors have been embedded in public water, electricity, oil, gas, buildings, transportation and other public service resources to form the Internet of Things. Using information and communication technologies, we will sense the core systems of urban operations, such as hydropower, communications, government affairs, and energy. Moreover, we can use computers to develop and analyze big data in municipal services. As a result, we can timely transmit, integrate, exchange, and use various types of urban public information, such as economy, culture, public resources, management services, citizen life, and ecological environment. Smart cities can improve the ability of interconnecting things and people, and help people fully develop and utilize information, to achieve a subtle, dynamic, and efficient resource allocation of urban. Smart city, a new urban development model, will make urban development more comprehensive, coordinated, and sustainable, and will make urban life healthier, more harmonious, and better.

### 2.2. Green Total Factor Productivity (GTFP)

GTFP is an indicator obtained by adding some factors such as environment and energy to TFP. GTFP can more comprehensively reflect the quality of national economic growth from the aspects of environmental protection and rational use of resources. In recent years, kinds of literature have reported the factors affecting GTFP [30,31,32].

There are three common types of measurement methods on total factor productivity as follows: Solow residual method (SRM), stochastic frontier production function method (SFA), and data envelopment analysis (DEA). The SRM [33] was proposed by Solow, who established a neoclassical production function $Y_{t}=A_{t}K_{t}^{\alpha }L_{t}^{\beta }$. However, the SRM has a disadvantage that it must satisfy the two assumptions of perfect competition and profit maximization. With an econometric method to measure total factor productivity, the SFA divides the total production function into the frontier production function and the non-efficiency part [34,35]. Though the SFA can overcome the shortcomings of the SRM, it shows another weakness, such as requiring a particular functional relationship and only confining to performance assessment of single-input-multi-output (MISO) production process. The DEA is a data-driven approach that does not require a defined production function. By combining the directional distance function (SBM) and the productivity change index, the DEA can be reconstructed [36] to measure GTFP that incorporates environmental pollution emissions as undesired outputs into the accounting system.

The measurement of GTFP includes two types of elements: Input factors and output factors. The input factors include labor input, energy input, and capital investment. The output factors include expected output and unexpected output. The expected output is generally measured in terms of GDP, and the unexpected output is the environmental output that measures the degree of environmental pollution.

### 2.3. Analysis of the Impact of Smart City Policy on GTFP

A smart city is an efficient and technologically advanced emerging city that combines green ecological development and economic development [37]. Since the concept of a smart city has been raised, it has always been closely related to the real problems faced by urban development. Green economy development is one of the most critical goals in implementing smart city policies. GTFP is an important indicator that combines economic growth with environmental protection to measure the quality of economic development. Therefore, we believe that smart city policy will have an impact on GTFP.

On the one hand, the smart city policy is conducive to optimizing and upgrading of industrial structure, promoting the efficiency of resource allocation, thereby reducing energy waste and improving GTFP. Under the smart city policy, the industrial structure achieves a transformation of industrial structural factors from labor-intensive to capital-intensive, and then from capital-intensive to technical-intensive [38]. Smart cities rely on Internet of Things, cloud computing, data mining, and other emerging information technologies and are mainly based on factors such as knowledge and technology. So that smart city policies can drive some development, such as information technologies, research and development (R&D), design, software, and other productive services. Then the industrial structure is optimized and upgraded. The smart city can sense urban development and changes in real time, intelligently produce and process big data, so that each agent can grasp accurate information and make scientific decisions [39]. Moreover, the smart city policy combines with existing technologies in sectors of transportation and energy to form a new business model [40]. For example, companies can intelligently monitor real-time monitoring, master market demand, and flexibly dispatch production factors, such as corporate capital, labor, and energy. For each decision-making agent, the smart city policy is conducive to adequately scheduling and adjusting human, material, financial and other resources based on the information available, continuously improving the efficiency and utilization of resource allocation, reducing or avoiding waste of resources, thereby improving GTFP.

On the other hand, the smart city policy promotes the development of advanced monitoring and governance technologies, monitoring pollutants and pollution sources in real time, reduce urban environmental pollution [21], and improve GTFP. The smart city construction optimizes and upgrades pollution control modes and technical methods by intelligent monitoring equipment. These devices can dynamically collect information from various environmental resources such as the atmosphere and water, and improve the monitor level of environmental pollution and the ability to acquire pollution information. Tele-monitoring technology is used to monitor the current distribution of lawns, public lands, and green belts [41]. It can effectively control various pollutants and pollution sources, promote environmental protection, and thus improve GTFP.

In summary, as an efficient, technologically advanced, green, and socially inclusive city [42], the smart city can be assessed by the GTFP to a certain extent. On the other hand, smart city policies can have an impact on GTFP.

## 3. Materials and Methods

### 3.1. Data

Our paper uses the data of China’s prefecture-level cities and removes the following unqualified urban samples: (a) Provinces, municipalities or autonomous regions that are smart city pilots, such as Hainan Province, Beijing, Shanghai, Xinjiang and Uygur Autonomous Region; (b) prefecture-level cities with administrative changes within the study period, such as Chaohu City, Bijie City, Tongren City, etc.; (c) prefecture-level cities with severe data loss, such as Lhasa City; and (d) prefecture-level cities containing counties or districts that are smart city pilots, such as Tangshan City and Handan City. The first batch of smart cities that emerged in 2012 was not eligible, so the second batch of smart cities that emerged in 2013 was regarded as the experimental group, and other cities in the provinces where the pilot cities were located were regarded as the control group. The third batch of pilot cities that appeared in 2014 was also removed but later used to test the robustness of the results. Finally, this paper selects the panel data of 200 cities in China from 2007–2016 as the research sample. The data are mainly from the China Statistical Yearbook (2006–2016), Shandong Statistical Yearbook (2006–2016), China Environmental Statistics Yearbook (2006–2016), the National Bureau of Statistics and provincial statistical bureaus, and missing data are estimated by the annual average growth rate method. For the specific experimental group and control group sample, see Table A1.

### 3.2. Measures

#### 3.2.1. Dependent Variable

The explanatory variable of this paper is green TFP. Referring to the practice of Fare et al. [43], our paper combined Malmquist’s TFP index and data envelopment analysis theory. In addition, we use Tone’s non-radial and non-angle slack-based measure (SBM) efficiency model [44] based on slack variables containing undesired output to construct the Malmquist productivity change index of intertemporal changes. The SBM efficiency model formula is as follows:

(1) $$\begin{array}{l} \min\rho^{*}=\frac{1+\frac{1}{t_{x}}\sum_{i=1}^{t_{x}}\frac{s_{i}^{x}}{x_{ik}}}{1-\frac{1}{t_{y}+t_{b}}(\sum_{r=1}^{t_{y}}\frac{s_{r}^{y}}{y_{rk}}+\sum_{l=1}^{t_{b}}\frac{s_{l}^{b}}{b_{lk}})}\\ s.t.\{\begin{array}{l} x_{ik}\geq \overset{n}{\underset{j=1,j\neq k}{\sum }}x_{ij}\lambda_{j}-s_{i}^{x}\\ b_{lk}\geq \overset{n}{\underset{j=1,j\neq k}{\sum }}b_{lj}\lambda_{j}-s_{l}^{b}\\ 1-\frac{1}{t_{y}+t_{b}}(\overset{t_{y}}{\underset{r=1}{\sum }}\frac{s_{r}^{y}}{y_{rk}}+\overset{t_{b}}{\underset{l=1}{\sum }}\frac{s_{l}^{b}}{b_{lk}})>0\\ s^{x},s^{y},s^{b},\lambda \geq 0;j=1,2,\ldots \ldots ,n\left(j\neq k\right) \end{array} \end{array}$$

where λ is the weight vector, $x_{ik}$, $y_{rk}$, $b_{lk}$ represent the input variable, the expected output, and the undesired output, respectively. $s_{}^{x}$, $s_{}^{y}$ and $s_{}^{b}$ represent the slack variables of the respective three types of elements, where $t_{x}$, $t_{y}$ and $t_{b}$ represent the three types of elements.

The Malmquist TFP index model is as follows:

(2) $$GTFP_{t}^{t+1}={\left[\frac{D^{t}\left(x^{t+1},y^{t+1},b^{t+1}\right)}{D^{t}\left(x^{t},y^{t},b^{t}\right)}\times \frac{D^{t+1}\left(x^{t+1},y^{t+1},b^{t+1}\right)}{D^{t+1}\left(x^{t},y^{t},b^{t}\right)}\right]}^{\frac{1}{2}}$$

where $GTFP_{t}^{t+1}$ represents the total factor productivity change index. If the value is greater than 1, the GTFP increases, and if it is less than 1, the GTFP decreases. Thus, the obtained Malmquist index reflects the growth of GTFP. Drawing on the practice of Chen et al. [45], this paper assumes that the green total factor growth rate of the base period is 1, and the annual multiplication of the Malmquist index represents the annual GTFP.

The input indicators selected in this paper include the three elements of capital, labor, and energy. We use the classic perpetual inventory method to measure capital investment. The basic formula is as follows:

(3) $$K_{t}=I_{t}+\left(1-\delta \right)K_{t-1}$$

where $K_{t}$ and $K_{t-1}$ represent the capital stock of period *t* and period *t* − 1, respectively, and δ represents the capital depreciation rate. Based on the practice of Zhang et al. [46], the fixed investment amount of each city in 2006 is defined at 10% as the initial capital stock, and the urban fixed assets depreciation rate δ is set to 9.6% in this article. Labor input is expressed by the number of employed people at the end of the year, and energy input is expressed by the total electricity consumption. Regarding output, this paper selects the GDP of each city as the desirable output and uses the entropy method to obtain the comprehensive index of environmental pollution based on the total amount of wastewater discharge, sulfur dioxide emissions, and solid waste production as undesired output.

#### 3.2.2. Key Explanatory Variable

In this paper, the key explanatory variable is smart city policies represented by du×dt. du is a dummy variable: If i is a smart city, it belongs to the treated group, and the corresponding du takes a value of 1. If i is not a smart city, then it belongs to the control group, and the corresponding du takes the value 0. dt is a dummy variable for policy implementation: Before the policy implementation, dt takes a value of 0, and after the policy is implemented, dt takes a value of 1.

#### 3.2.3. Control Variable

The control variables of this paper include the level of economic development (ECO), the degree of government intervention (GOV), industrial structure (IND), traffic conditions (TRAF), and technological innovation (TECH). The level of economic development is expressed by per-capita GDP, the government intervention capacity is expressed by the proportion of fiscal expenditure to GDP, the traffic condition is expressed by the per-capita road area, and the industrial structure is expressed by the ratio of secondary industry to tertiary industry.

### 3.3. The Estimation Model

The PSM method was first proposed by Rosenbaum and Rubin [47] in 1983. It is a statistical method for dealing with sample selectivity bias. This method uses a calculated propensity score to find one or more individuals with characteristics that are the same or similar to those of each individual in the treated group and to take them as the control group. This paper uses the kernel matching method to determine the control group.

The DID method has become increasingly popular in policy analysis [48]. It studies the differential effect of a policy that does not affect everybody at the same time and in the same way on a treated group versus a control group [49]. The DID model of this paper is set as follows:

(4) $$lnGTFP_{it}=\alpha_{0}+\alpha_{1}du\times dt+\overset{N}{\underset{i=1}{\sum }}\beta_{i}X_{it}+\varepsilon_{it}$$

where du×dt is an interaction term between a group dummy variable and a policy dummy variable, and the coefficient α1 reflects the net effect of policy implementation. X is a series of control variables, including economic development level (ECO), government intervention capability (GOV), industrial structure (TECH), traffic conditions (TRAF) and industrial structure (IND).

## 4. Analysis and Results

In this paper, we use statistical software Stata 15.0 (Stata Corp, College Station, Tex., USA) to implement analysis and illustrate table or graphic results.

### 4.1. Descriptive Statistics

Descriptive statistics were captured on the main variables of the treated group, the control group, and the whole sample. The sample size, mean and standard deviation are shown in Table 1, and the GTFP of the treated group is significantly higher than that of the control group (the mean of GTFP is 0.592 in the treated group and is 0.406 in the control group). This result is a preliminary indication that smart city policies can promote urban GTFP.

### 4.2. Regression Analysis

We assess the impact of smart city policies on GTFP by using linear regression analysis, and the results are shown in Table 2. Model 1 contains only the key explanatory variable (GTFP), and the coefficient is significantly positive, indicating that smart city policies have a positive impact on green total factor productivity. On the basis of Model 1, Models 2–6 are added to the control variables one by one. It is clear that the coefficient of GTFP remains positive, and further supporting the earlier conclusions.

### 4.3. Robustness Test

#### 4.3.1. Robustness Test Based on PSM-DID Method

To eliminate the influence of sample selection bias, we should select those non-smart cities whose characteristics are similar to those of the treated group as the control group. In this paper, the PSM method is used to perform logit regression on the control variables, and a control group is determined based on those cities whose propensity score is closest to that of the treated group is determined. The results are shown in Table 3. Before implementing the “smart city”, the GTFPs of the treated group and the control group are 0.435 and 0.383, respectively. The average treatment effect on the treated (ATT) is 0.052, which is significant at the 1% level. After the policy, the GTFP is 0.725 in the treated group and is 0.594 in the control group, and the ATT is 0.131. At the same time, the result of the difference-in-differences is also significant at the 1% level. Obviously, the results passed the robustness test.

**Figure 1:**
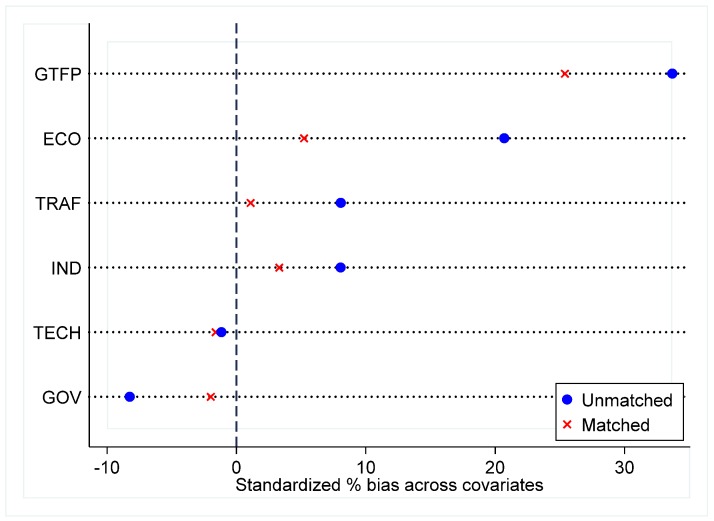
The standard deviation before and after matching the dependent and control variables.

Before using the PSM-DID method, a joint support hypothesis test is necessary to prove its applicability. As shown in Table 4, the control variables (ECO, TECH, GOV, IND, and TRAF) are all significantly different between the treated group and the control group before matching but are not significant after matching. The dependent variable (GTFP) is significantly different before and after matching. To see the differences between matched and unmatched samples more intuitively, Figure 1 indicates the standard deviation before and after matching the dependent variable and control variables. The results show that the joint support hypothesis test is passed.

**Figure 2:**
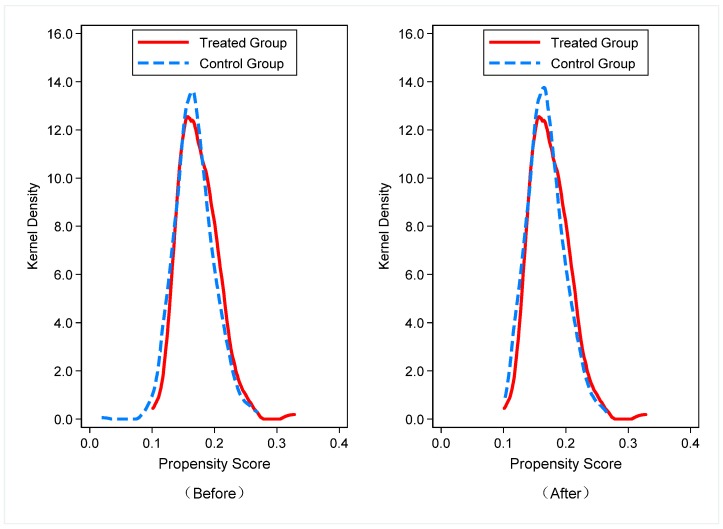
The probability density of the propensity scores.

Before the regression analysis, we tested the matching effect between the treated and control groups using the kernel density propensity matching method (see Figure 2). The probability density of the propensity scores of the treated and control groups after matching has essentially been consistent, indicating that the matching effect is better, which further proves the applicability and reasoning for the use of PSM-DID in this paper.

#### 4.3.2. Changing the Treated Group

We now offer a further robustness test using the third batch of smart cities that emerged in 2014 as the new treated group. As before, we implement a regression analysis and obtain some valuable results, as shown in Table 5. After changing the treated group, smart city policies still play a significant role in promoting GTFP.

#### 4.3.3. Further Testing

**Figure 3:**
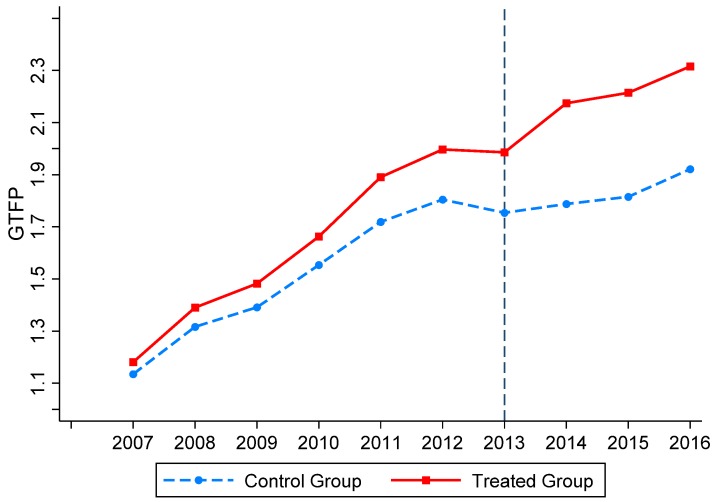
Trends in GTFP of the treated and control groups.

To determine whether the increase in GTFP is affected by “smart city” or other policies, Figure 3 shows the trend in the GTFP of the treated and control groups. It can be seen in Figure 2 that before 2013 (when the “smart city” was implemented), the trends of the treated and control groups remain basically the same. After the implementation of the policy, the GTFP gap between the two groups significantly widened, providing further support for the conclusions of this paper.

### 4.4. Heterogeneity Analysis

Table 6 shows that it is significant in promoting GTFP regardless of medium-sized cities or large cities. However, the larger the city size, the higher the promotion of smart city policy to GTFP, and the more significant the promotion effect. This may be because the information development level in medium-sized cities is weaker than that in large cities. It also shows that the urban problems in the development of traditional cities, such as congestion and environmental pollution, may be mitigated after the reform of the governance model and the deepening of urban innovation.

The above results show that the smart city policy can significantly improve the GTFP. Next, we will further answer the following two questions: Does the GTFP increase significantly for different city sizes? Is there a difference in the degree of improvement?

As we know, compared with small cities, larger cities have more complete functions, stronger innovation capabilities, and higher resource allocation, and utilization efficiency. However, overpopulation will lead to urban congestion and more serious environmental pollution. The smart city policy can not only improve the efficiency of urban resource allocation but also solve urban problems by technological innovation. As a result, GTFP has also be improved. So it is necessary for us to further verify the impact of GTFP on cities of different sizes. The division of the city in this paper is based on the latest standard in the “Notice on Adjusting the Dividing Standards of Urban Size” issued by the State Council in 2014. Due to the limited sample size, there are fewer small-scale cities, resulting in unreliable regression results, so only the results of cities above medium-sized scale are reported. The regression results are shown in Table 6.

## 5. Discussion

From an empirical perspective, we take the panel data of 200 cities in China from 2007–2016 as the research object and regards the smart city policy as a quasi-natural experiment, using the PSM-DID method to estimate the impact of “smart city” policies on GTFP. From the regression results, the smart city policy has a significant positive effect on GTFP. To verify the result accuracy, the robustness test is carried out by jointly supporting the hypothesis test and changing the control group. These tests have passed. Ultimately, we conclude that smart city policies can significantly improve urban GTFP.

To enrich research conclusions, we analyze the heterogeneity of urban scale to test whether the impact of different city-scale smart city policies on GTFP is different. Regression results indicate that the larger the city size, the higher the promotion of smart city policy to GTFP, and the more significant the promotion effect. It may be because the larger the city, the more complete the city’s functions, and the better the conditions for implementing the smart city policy.

Our contribution includes two aspects. First, from the perspective of the urban development pattern, we offer the first study of the impact of smart city policies on GTFP. It is a theoretical supplement to smart city policies and enriches the research on the factors affecting GTFP. Second, the resource and environmental problems stemming from the process of economic development appear not only in China but also in other countries. The environmental problems in developing countries have received very little attention. At present, research on GTFP is mainly concentrated on China. Certainly, this paper will also be useful for other countries.

## 6. Conclusions

With a deep expansion and integration of information technology and the Internet of Things, smart cities are realizing intelligence in transportation, energy utilization, resource allocation, and urban pollution discharge through a large number of innovative technologies. As a new urban development pattern, smart cities establish operational command centers and use the Internet of Things technologies (such as intelligent transportation and modern information communication) to collect information and analyze data. The green concept and advanced technology are perfectly integrated into urban planning, which promotes the improvement of urban resource allocation efficiency [20], the upgrading of industrial structure and the improvement of innovation capability [19], thereby ultimately promoting the transformation of urban production and lifestyle and promoting the development of a green economy.

In addition, the city heterogeneity analysis shows that the key to the city development is not its large scale, but whether the model of the city management and development pattern is innovative. Therefore, in the process of implementing the new urbanization strategy, the introduction of smart technology is conducive to improving urban innovation capacity and resource allocation efficiency and reducing pollution. On the one hand, big cities give full play to their powerful urban functions and vigorously develop smart technologies. Therefore, we should apply advanced information technology to all aspects of production and life to alleviate and eliminate urban problems. On the other hand, small and medium-sized cities make full use of the advantages of smart city policies to develop smart projects that are suitable for the characteristics of the city and maximize urban governance and operational efficiency.

There are some implications, limitations, and future directions: (a) Due to the limited sample size, the potential limitation may affect regression results, so we can extend the evidence scope from China to the whole world in order to show spatiotemporal effects; (b) the smart city policies can significantly improve urban GTFP but it does not mean that we need to invest as much as possible in smart cities, so we can pay attention to the appropriate investment scale and the crowding-out effect of smart city policies; (c) we should pay attention to spillover and siphon effects in smart city construction.

## Figures and Tables

**Table 1:** Description of main variables.

Variable	Group	*n*	Mean	SD
GTFP	Treated Group	300	0.551	0.339
	Control Group	1480	0.441	0.309
	Total Sample	1780	0.460	0.317
ECO	Treated Group	300	10.372	0.669
	Control Group	1480	10.235	0.647
	Total Sample	1780	10.258	0.652
TECH	Treated Group	300	−4.694	0.675
	Control Group	1480	−4.696	0.741
	Total Sample	1780	−4.688	0.730
IND	Treated Group	300	0.372	0.469
	Control Group	1480	0.337	0.405
	Total Sample	1780	0.343	0.417
TRAF	Treated Group	300	2.340	0.839
	Control Group	1480	2.181	0.590
	Total Sample	1780	2.191	0.639
GOV	Treated Group	300	−1.815	0.483
	Control Group	1480	−1.774	0.501
	Total Sample	1780	−1.781	0.498

Notes: SD— represents standard deviations, *n*—represents the number of samples.

**Table 2:** Regression results for the impact of smart city policies on green total factor productivity (GTFP).

Variable	Model 1	Model 2	Model 3	Model 4	Model 5	Model 6
du×dt	0.285 ***	0.179 ***	0.165 ***	0.176 ***	0.176 ***	0.175 ***
	(0.03)	(0.03)	(0.03)	(0.03)	(0.03)	(0.03)
ECO	-	0.221 ***	0.248 ***	0.237 ***	0.248 ***	0.248 ***
	-	(0.01)	(0.01)	(0.01)	(0.01)	(0.01)
TECH	-	-	−0.048 ***	−0.049 **	−0.047 ***	−0.046 ***
	-	-	(0.01)	(0.01)	(0.01)	(0.01)
IND	-	-	-	0.067 ***	0.065 ***	0.087 ***
	-	-	-	(0.02)	(0.02)	(0.02)
TRAF	-	-	-	-	−0.021 ***	−0.020 *
	-	-	-	-	(0.01)	(0.01)
GOV	-	-	-	-	-	−0.006
	-	-	-	-	-	(0.02)
_cons	0.441 ***	−1.820 ***	−2.318 ***	−2.234 ***	−2.294 ***	−2.284 ***
	(0.01)	(0.10)	(0.15)	(0.15)	(0.15)	(0.15)
*n*	1780	1780	1780	1780	1780	1780

Notes: Standard deviations are in parentheses, *, ** and *** indicate significant differences at *p* < 0.01, *p* < 0.05 and *p* < 0.001, respectively. _cons—represents a constant term, *n*—represents the number of samples.

**Table 3:** Robustness test based on the difference-in-differences propensity score matching (PSM-DID) method.

	GTFP	SE	|*t*|	*p* > |*t*|
Before				
Control	0.383			
Treated	0.435			
Diff	0.052	0.017	3.06	0.002 ***
After				
Control	0.594			
Treated	0.725			
Diff	0.131	0.021	6.24	0.000 ***
Diff-in-Diff	0.079	0.027	2.91	0.004 **

Notes: *, ** and *** indicate significant differences at *p* < 0.01, *p* < 0.05 and *p* < 0.001, respectively. SE—represents standard error.

**Table 4:** Jointly support the hypothesis test.

Variable	Unmatched/Matched	Mean		*t*	*p* > |*t*|
		Treated Group	Control Group		
ECO	U	10.372	10.235	3.31	0.001 ***
	M	10.352	10.318	0.68	0.496
TECH	U	−4.694	−4.686	−0.18	0.859
	M	−4.688	−4.677	−0.20	0.844
GOV	U	−1.815	−1.774	−1.29	0.198
	M	−1.811	−1.801	−0.25	0.804
IND	U	0.372	0.337	1.34	0.181
	M	0.372	0.357	0.41	0.685
TRAF	U	2.240	2.181	1.45	0.148
	M	2.215	2.207	0.14	0.891
GTFP	U	0.551	0.442	5.49	0.000 ***
	M	0.550	0.467	3.16	0.002 ***

Notes: *, ** and *** indicate significant differences at *p* < 0.01, *p* < 0.05 and *p* < 0.001, respectively.

**Table 5:** Regression results after changing the treated group.

Variable	Model 7	Model 8	Model 9	Model 10	Model 11	Model 12
du×dt	0.177 ***	0.147 ***	0.149 ***	0.160 ***	0.164 ***	0.158 ***
	(0.03)	(0.03)	(0.03)	(0.03)	(0.03)	(0.03)
ECO	-	0.214 ***	0.240 ***	0.230 ***	0.245 ***	0.247 ***
	-	(0.01)	(0.01)	(0.01)	(0.01)	(0.01)
TECH	-	-	−0.046 ***	−0.048 ***	−0.045 ***	−0.040 ***
	-	-	(0.01)	(0.01)	(0.01)	(0.02)
IND	-	-	-	0.081 ***	0.077 ***	0.086 ***
	-	-	-	(0.02)	(0.02)	(0.02)
TRAF	-	-	-	-	−0.032 **	−0.032 **
	-	-	-	-	(0.01)	(0.01)
GOV	-	-	-	-	-	0.025
	-	-	-	-	-	(0.02)
_cons	−0.439 ***	−1.742 ***	−2.224 ***	−2.157 ***	−2.222 ***	−2.190 ***
	(0.01)	(0.10)	(0.14)	(0.14)	(0.14)	(0.15)
*n*	1700	1700	1700	1700	1700	1700

Notes: Standard deviations are in parentheses, *, ** and *** indicate significant differences at *p* < 0.01, *p* < 0.05 and *p* < 0.001, respectively.

**Table 6:** Regression results of different city sizes.

	Medium City	Large City	Megacity
du×dt	0.103 **	0.145 ***	0.169 ***
	(0.05)	(0.03)	(0.03)
ECO	0.329 ***	0.316 ***	0.215 ***
	(0.03)	(0.02)	(0.02)
TECH	−0.0240	−0.107 ***	−0.031 **
	(0.02)	(0.01)	(0.02)
IND	0.0470	0.087 ***	0.0260
	(0.03)	(0.02)	(0.03)
TRAF	−0.079 ***	−0.0100	−0.00400
	(0.02)	(0.02)	(0.02)
GOV	−0.075 ***	0.171 ***	0.181 ***
	(0.03)	(0.02)	(0.02)
_cons	−3.049 ***	−2.932 ***	−1.496 ***
	(0.28)	(0.23)	(0.21)
*n*	797	491	406

Notes: Standard deviations are in parentheses, *, ** and *** indicate significant differences at *p* < 0.01, *p* < 0.05 and *p* < 0.001, respectively.

## Appendix A

**Table A1:** Distribution of smart cities.

	Treated Group	Control Group
2013	Shanxi Province (2), Inner Mongolia Autonomous Region (2), Liaoning Province (1), Jilin Province (1), Shandong Province (1), Heilongjiang Province (2), Sichuan Province (2), Ningxia Hui Autonomous Region (1), Anhui Province (2), Guangdong Province (2), Shaanxi Province (3), Guangxi Zhuang Autonomous Region (3), Jiangsu Province (1), Henan Province (1), Hubei Province (2), Fujian Province (2), Gansu province (4)	Shanxi Province (5), Liaoning Province (11), Heilongjiang Province (7), Zhejiang Province (8), Shandong Province (8), Yunnan Province (6), Inner Mongolia Autonomous Region (5), Jilin Province (2), Sichuan Province (11), Anhui Province (6), Shaanxi Province (5), Guangdong Province (14), Guangxi Zhuang Autonomous Region (7), Jiangsu Province (3), Hebei Province (5), Henan Province (13), Hubei Province (7), Hunan Province (6), Gansu province (6), Ningxia Hui Autonomous Region (2), Fujian Province (10), Qinghai Province (1)
2014	Shanxi Province (1), Inner Mongolia Autonomous Region (1), Jilin Province (1), Anhui Province (3), Fujian Province (2), Shandong Province (1), Henan Province (2), Hubei Province (1), Guangxi Zhuang Autonomous Region (2), Sichuan Province (3), Yunnan Province (1), Shaanxi Province (1), Gansu province (2), Ningxia Hui Autonomous Region (1)	Shanxi Province (5), Liaoning Province (11), Heilongjiang Province (7), Zhejiang Province (8), Shandong Province (8), Yunnan Province (6), Inner Mongolia Autonomous Region (5), Jilin Province (2), Sichuan Province (11), Anhui Province (6), Shaanxi Province (5), Guangdong Province (14), Guangxi Zhuang Autonomous Region (7), Jiangsu Province (3), Hebei Province (5), Henan Province (13), Hubei Province (7), Hunan Province (6), Gansu province (6), Ningxia Hui Autonomous Region (2), Fujian Province (10), Qinghai Province (1)
